# Evolution of ceftazidime–avibactam resistance driven by mutations in double-copy *bla*_KPC-2_ to *bla*_KPC-189_ during treatment of ST11 carbapenem-resistant *Klebsiella pneumoniae*

**DOI:** 10.1128/msystems.00722-24

**Published:** 2024-09-17

**Authors:** Xiaofan Zhang, Yinrong Xie, Ying Zhang, Tailong Lei, Longjie Zhou, Jiayao Yao, Lin Liu, Haiyang Liu, Jintao He, Yunsong Yu, Yuexing Tu, Xi Li

**Affiliations:** 1 Centre of Laboratory Medicine, Zhejiang Provincial People’s Hospital, People’s Hospital of Hangzhou Medical College, Hangzhou, Zhejiang, China; 2 Department of Clinical Laboratory, Feicheng Hospital of Traditional Chinese Medicine, Feicheng, Shandong, China; 3 Department of Clinical Laboratory, Xiamen Hospital of Traditional Chinese Medicine, Xiamen, Fujian, China; 4 Department of Infectious Diseases, Sir Run Run Shaw Hospital, Zhejiang University School of Medicine, Hangzhou, Zhejiang, China; 5 Center for General Practice Medicine, Department of Infectious Diseases, Zhejiang Provincial People’s Hospital, Affiliated People’s Hospital, Hangzhou Medical College, Hangzhou, Zhejiang, China; 6 Department of Critical Care Medicine, Tongde Hospital of Zhejiang Province, 234 Gucui Road, Hangzhou, Zhejiang, China; Third Institute of Oceanography Ministry of Natural Resources, Xiamen, China

**Keywords:** ST11, CRKP, *bla*_KPC-189_, double-copy, CZA resistance

## Abstract

*Klebsiella pneumoniae* carbapenemase (KPC) variants can contribute to resistance to ceftazidime–avibactam (CZA) in *Klebsiella pneumoniae* (KP). However, two-copy KPC variant-mediated resistance to CZA has rarely been reported to date. Here, we aimed to clarify the evolutionary trajectory of CZA resistance driven by mutations in double-copy *bla*_KPC-2_ to *bla*_KPC-189_ carried by the tandem core structure (IS*Kpn6-bla*_KPC_-IS*Kpn27-tnpR*-IS*26*) during treatment of ST11 carbapenem-resistant *K. pneumoniae* (CRKP). The CZA-resistant KP strain carried double-copy *bla*_KPC-189_, a variant with alanine–threonine and aspartate–tyrosine substitutions at Ambler amino acid positions 172 (A172T) and 179 (D179Y) of *bla*_KPC-2_. Clone experiments confirmed that, compared with that of the wild-type *bla*_KPC-2_ clone strain, the minimum inhibitory concentration of CZA increased 16-fold in the *bla*_KPC-189_-mutant strain. Furthermore, protein structure analysis revealed the A172T and D179Y mutations of *bla*_KPC-189_ can have a direct effect on the binding affinity of CAZ and AVI for KPC. Sequence comparison revealed that *bla*_KPC-189_ was mutated in a double-copy format upon CZA exposure, which was carried by the IS*26*-mediated tandem core structure IS*Kpn27-bla*_KPC_-IS*Kpn6*. This tandem core structure apparently evolves *in vivo* during infection, although not by self-transferring, and multiple IS*Kpn27-bla*_KPC_-IS*Kpn6* copy numbers could mediate transferable CZA resistance upon mobilization. In addition, compared with the wild-type *bla*_KPC-2_ gene, the *bla*_KPC-189_ gene had no fitness cost. In summary, our study highlighted the emergence of CZA-resistant *bla*_KPC-189_ variants in the ST11 clone and the presence of a double-copy *bla*_KPC-189_ in the IncFII-type plasmid, which is carried by a tandem core structure (IS*26-*IS*Kpn6-bla*_KPC-189_-IS*Kpn27-tnpR*-IS*26*).

**IMPORTANCE:**

To date, ceftazidime–avibactam (CZA) resistance caused by double-copy *Klebsiella pneumoniae* carbapenemase (KPC) variants has not been elucidated. The multicopy forms of carbapenem resistance genes carried by the same plasmid are relatively rare in most carbapenem-resistant *Enterobacteriaceae*. In this study, we elucidate the evolutionary trajectory of CZA resistance in ST11 carbapenem-resistant *K. pneumoniae* harboring a double-copy blaKPC and provide new insights into the mechanisms of acquired resistance to CZA.

## INTRODUCTION

*K. pneumoniae* is an opportunistic pathogen that transforms into carbapenem-resistant *K. pneumoniae* (CRKP) upon the acquisition of plasmids carrying carbapenem resistance genes, a superbug that causes hard-to-treat infections and poses a significant threat to human health (1). Various causes contribute to carbapenem resistance, and the presence of carbapenemases is the major mechanism of carbapenem antibiotic resistance in CRKP strains (2). A global study indicated that *K. pneumoniae* carbapenemase (KPC), metallo-β-lactamases (MBLs), and OXA-48-like carbapenemases were the primary enzyme types produced by CRKP (3). In particular, class A KPC-type serine carbapenemases are consistently detected at high rates (3, 4). The vast majority of KPC-producing *K. pneumoniae* (KPC-KP) isolates belonged to clonal complex 258 (CG258), among which ST11 was the predominant sequence type and was the major hospital-acquired clone in China (4, 5). In addition, the epidemic of ST11-type CRKP isolates is due to their capacity to harbor KPC-producing plasmids, which cause resistance to antimicrobial drugs and enhance their survival (5).

In recent years, the novel β-lactamase inhibitor avibactam has been developed to treat KPC-KP strains and has been used in combination with ceftazidime in clinical therapy (6). The ceftazidime–avibactam (CZA) combination exhibited excellent *in vitro* activity against KPC-carrying *Enterobacteriaceae* and was associated with decreased death rates in clinical patients (7). However, since the widespread use of CZA, numerous KPC variants related to resistance after treatment with CZA have been identified, suggesting the high evolutionary potential of this enzyme (8). Therefore, CZA-resistant KPC-KP isolates are frequently detected in patients treated with CAZ-AVI (9, 10). CZA resistance in KP strains has been described to be associated with different resistance mechanisms, such as various mutations in *bla*_KPC_ and *bla*_CTX-M_ and the overexpression of the AcrAB-TolC efflux system (11). Among them, mutations in *bla*_KPC_ were the most common mechanism and caused resistance normally associated with the Ω-loop, which is a conserved motif present in class A β-lactamases comprising amino acid residues Arg164 to Asp179 (9, 10, 12, 13). Single amino acid substitutions within the Ω-loop of KPC may reduce the susceptibility of strains to CZA (9, 14, 15). In general, an increase in the copy number of *bla*_KPC-2_ on a plasmid increases bacterial resistance to β-lactam antibiotics (16); however, CZA resistance caused by double-copy KPC variants has thus far not been elucidated. In addition, in most carbapenem-resistant *Enterobacteriaceae* (CRE), the predominant form of carbapenemase resistance genes on the same plasmid is a single type or a single copy, whereas multicopy forms of carbapenem resistance genes carried by the same plasmid are relatively rare.

Here, we clarified the evolution of CZA resistance driven by a double-copy *bla*_KPC-2_ to *bla*_KPC-189_ mutation during treatment. A novel KPC variant (*bla*_KPC-189_, CZA-resistant) was identified. We elucidated the evolutionary trajectory of CZA resistance and provided new insights into the mechanism of acquired resistance to CZA.

## RESULTS

### Patient information and isolation characteristics

**Fig 1:**
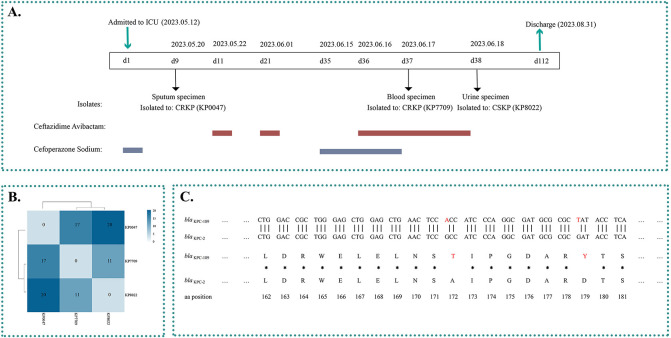
(A) Bacterial culture data and antibiotic treatment data for patients. (**B)** SNP difference heatmap of three *Klebsiella pneumoniae* strains. The numbers in the heatmap are the number of SNPs that varied between strains and are indicated by different colors. (**C)** Amplicon alignments between nucleotide and amino acid (aa) sequences of *bla*_KPC-189_ and *bla*_KPC-2_ surrounding the mutation. Two nucleotide mutations were identified in the *bla*_KPC-189_ gene compared to *bla*_KPC-2_, which resulted in mutations at amino acid positions 172 and 179 of the KPC-2 protein. The red letters represent mutant nucleotides and amino acids. Dotted line, common sequence; *, common amino acid; L, Leu; D, Asp; R, Arg; W, Trp; E, Glu; N, Asn; S, Ser; A, Ala; I, Ile; P, Pro; G, Gly.

The patient is an 82-year-old female who was hospitalized in May 2023 for unconsciousness, and she was diagnosed with septicopyemia, lung infection, and so on. During hospitalization, *K. pneumoniae* KP0047 (CZA-sensitive, *bla*_KPC-2_-positive) was isolated from sputum specimens of patient on 20 May 2023. After CZA treatment (1.25 g iv q8h), *K. pneumoniae* KP7709 (CZA-sensitive, *bla*_KPC-2_-positive), and KP8022 (CZA-resistant, *bla*_KPC-189_-positive) were isolated from blood and urine specimens on 17 June and 18 June 2023, respectively (Fig. 1A). Therefore, tigecycline and polymyxin were substituted for CZA treatment after isolation of CZA-R *K. pneumoniae* (CSKP, KP8022, *bla*_KPC-189_-positive). Notably, the KP7709 (CZA-sensitive) and KP8022 (CZA-resistant) strains were successively isolated during the final CZA treatment (1.25 g iv q8h) (Fig. 1A). Antibiotic susceptibility testing revealed that the CRKP isolates (KP0047 and KP7709) were resistant to most antibiotics, including cefepime, ceftazidime, ertapenem, imipenem, meropenem, ciprofloxacin, and amikacin, whereas they were susceptible to colistin, tigecycline, CZA, and CFDC. The resistance of the CSKP isolate (KP8022) was similar to that of the CRKP isolates, except for CZA resistance and recovery of imipenem and meropenem resistance (Table 1). In addition, compared to KP0047 and KP7709, the minimum inhibitory concentration (MIC) value of CFDC for KP8022 increased 16-fold (Table 1).

**TABLE 1:** Antibiotic susceptibility of the strains used in this study (mg/L)^*f*^

Isolates	FEP	CAZ	ETP	IPM	MEM	CIP	AMK	CST	TGC	CZA	CFDC
KP0047	>128	>128	>128	64	>128	32	>128	0.125	0.06	2	0.25
KP7709	>128	>128	>128	128	>128	16	>128	0.125	0.06	4	0.25
KP8022	>128	>128	64	0.5	4	16	>128	0.125	0.06	>128	4
*E. coli* DH5a	0.25	0.125	<0.06	0.125	<0.06	<0.06	0.25	<0.06	0.03	0.25	0.125
*E. coli* DH5a_pKP7709_KPC^*a*^	128	64	8	4	4	<0.06	>128	<0.06	0.25	0.5	0.25
*E. coli* DH5a_pKP8022_KPC^*b*^	64	128	<0.06	0.125	<0.06	<0.06	>128	<0.06	0.25	8	1
*E. coli* DH5a_pCR2.1^*c*^	0.25	0.125	<0.06	0.25	<0.06	<0.06	2	0.25	0.03	0.25	0.125
*E. coli* DH5a_pCR2.1_KPC-2^*d*^	128	>128	64	64	32	<0.06	2	<0.06	0.125	1	0.125
*E. coli* DH5a_pCR2.1_KPC-189^*e*^	16	>128	0.125	0.25	0.125	<0.06	0.5	<0.06	0.125	16	1

^*a*^ *E. coli* DH5a_pKP7709_KPC, *E. coli* DH5a was transformed by pKP7709_KPC plasmid carrying wild-type *bla*_KPC-2_ gene.

^*b*^ *E. coli* DH5a_pKP8022_KPC, *E. coli* DH5a was transformed by pKP8022_KPC plasmid carrying wild-type *bla*_KPC-189_ gene.

^*c*^ *E. coli* DH5a_pCR2.1, *E. coli* DH5a was transformed by expression plasmid pCR2.1-TOPO as a control.

^*d*^ *E. coli* DH5a_pCR2.1_KPC-2, *E. coli* DH5a was transformed by a pCR2.1_KPC-2 plasmid carrying wild-type *bla*_KPC-2_ gene.

^*e*^ *E. coli* DH5a_pCR2.1_KPC-189, *E. coli* DH5a was transformed by a pCR2.1_KPC-189 plasmid carrying *bla*_KPC-189_ gene.

^*f*^ FEP, cefepime; CAZ, ceftazidime; ETP, ertapenem; IPM, imipenem; MEM, meropenem; CIP, ciprofloxacin; AMK, amikacin; CST, colistin; TGC, tigecycline; CZA, ceftazidime–avibactam; CFDC, cefiderocol.

Whole-genome sequence analysis revealed that three ST11-type CRKP isolates harbored multiple resistance genes, including *bla*_SHV_, *bla*_CTX-M-176_, *fosA*6, *sul1*, *aadA2*, *bla*_TEM-1_, *catA2*, and *rmtB1*, as well as all carrying a variety of plasmids, including IncFII, IncR, and CoIRNAI (Table 2). Interestingly, both the KP0047 and KP7709 isolates were *bla*_KPC-2-_positive, while the KP8022 isolate was positive for the *bla*_KPC-189_ gene. The whole-genome sequence data revealed that KP8022 had less than 20 SNPs compared with the other clinically isolated strains KP7709 and KP0047, which indicated that these strains were highly homologous to each other (Fig. 1B). Among them, the smallest SNP difference existed between KP7709 and KP8022 (Fig. 1B). Moreover, continued exposure to CZA might lead to the evolution of CZA resistance in CRKP strains. We explored the mechanism of KP8022 resistance to CZA using KP7709 as a reference.

**TABLE 2:** Detailed information of the clinical strains used in this study

Isolates	MLST	Plasmid type	Antimicrobial resistance genes	Sample	Accession numbers
KP0047	ST11	IncFII, IncR, CoIRNAI	*bla*_SHV-158_ *, bla*_CTX-M-176_ *, fosA6, bla*_KPC-2_ *, sul1, aadA2, bla*_TEM-1_ *, catA2, rmtB1*	Human Sputum	JBCGZU000000000
KP7709	ST11	IncFII, IncR, CoIRNAI	*bla*_SHV-182_ *, bla*_CTX-M-176_ *, fosA6, bla*_KPC-2_ *, sul1, aadA2, bla*_TEM-1_ *, catA2, rmtB1*	Human Blood	JBAGBV000000000
KP8022	ST11	IncFII, IncR, CoIRNAI	*bla*_SHV-182_ *, sul1, aadA2, bla*_CTX-M-176_ *, fosA6, bla*_TEM-1_ *, bla*_CTX-M-15_ *, bla*_TEM-1_ *, rmtB1, bla*_KPC-189_ *, catA2*	Human Urine	CP144138, CP144139, CP144140 and CP144141

### Detection and prevalence of KPC variants

**Fig 2:**
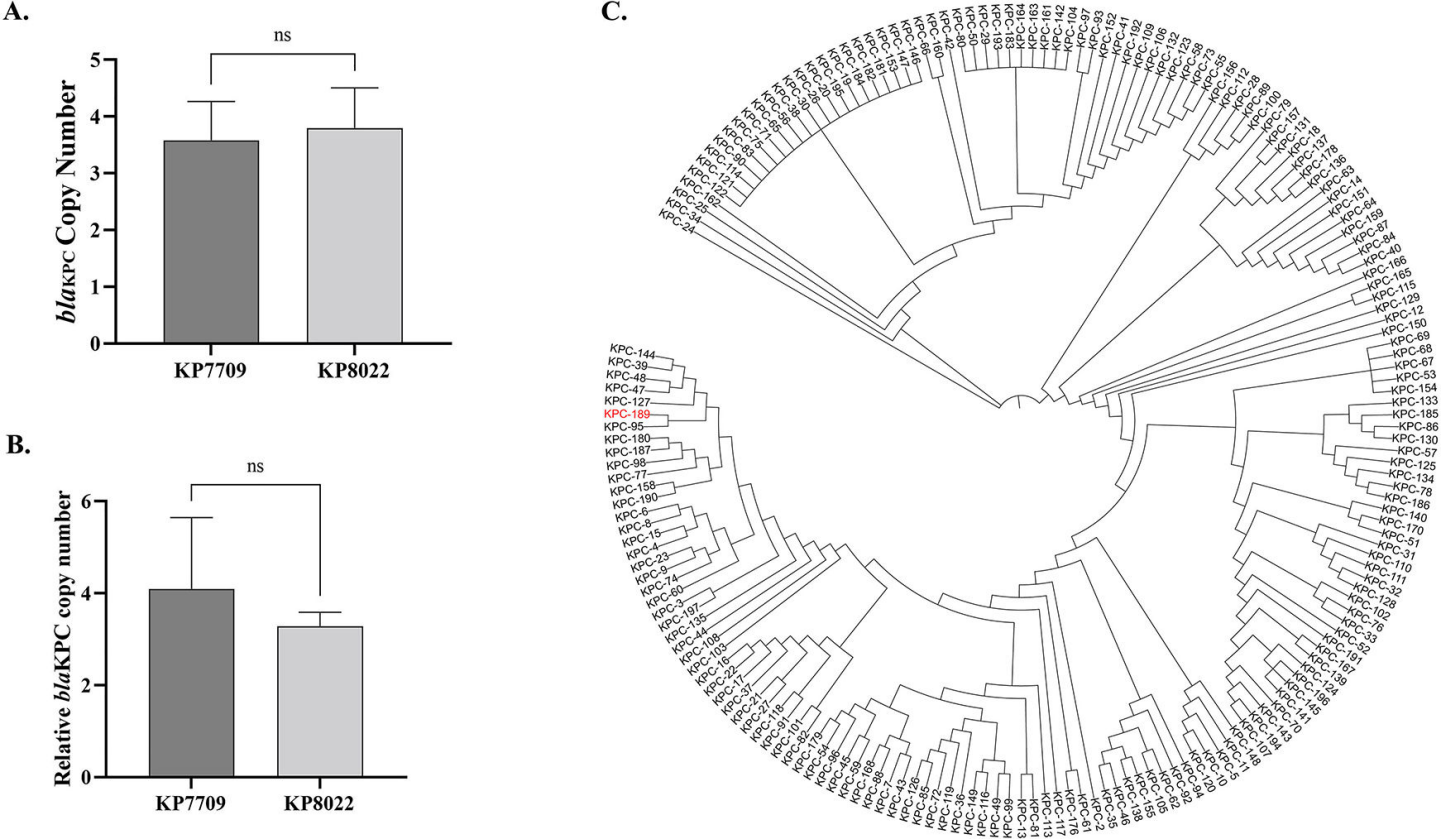
**(A) ***bla*_KPC_ gene copy number comparison among strains. (**B)** Relative quantification of *bla*_KPC_. *pgi* was used as a control. (**C)** Evolutionary tree analysis of KPC variants.

Comparative genomics revealed that different KPC variants were present in three *K. pneumoniae* isolates, which included a new KPC variant (*bla*_KPC-189_). The *bla*_KPC-189_ variant showed alanine–threonine and aspartate–tyrosine substitutions at amino acid positions 172 (A172T) and 179 (D179Y) (Fig. 1C). In addition, we found that KP8022 contained a double copy of *bla*_KPC-189_, and we hypothesized that KP7709 harbored two copies of the carbapenem resistance gene *bla*_KPC-2_, both of which were mutated to *bla*_KPC-189_ under the pressure of CZA. To confirm this hypothesis, we used real-time quantitative PCR (RT-qPCR) and sequencing depth to determine the number of copies of *bla*_KPC_ per cell, and the results confirmed that both the KP7709 and KP8022 isolates contained multiple copies of KPC (*bla*_KPC-2_ and *bla*_KPC-189_ were all >3 copies/cell) (Fig. 2A and B). In addition, a core genome phylogenetic tree was constructed, and the data showed that the variants were clustered in different clades according to different mutation sites (Fig. 2C).

### Association of *bla*_KPC-189_ with decreased susceptibility to CZA and CFDC

To investigate whether *bla*_KPC-189_ mediates CZA resistance and decreases CFDC susceptibility, a cloning experiment was performed with wild-type *bla*_KPC-2_ as a control. The effectiveness of the KPC strains carried by each cloned strain was determined by measuring the susceptibility of the strains to antibiotics. The results showed that the MIC of CZA in *Escherichia coli* DH5a_pCR2.1_KPC-189 increased from 1 to 16 mg/L compared with that of the cloned strain carrying wild-type *bla*_KPC-2_, confirming that *bla*_KPC-189_ plays an important role in mediating CZA resistance (Table 1). In addition, the MIC value of CFDC in *E. coli* DH5a_pCR2.1_KPC-189 increased eightfold compared with the *E. coli* DH5a_pCR2.1_KPC-2, which indicated that KPC-189 reduced the CFDC susceptibility of the strain (Table 1). Moreover, the *E. coli* DH5a_pCR2.1_KPC-189 clone strain also recovered susceptibility to carbapenem antibiotics, including ertapenem, imipenem, and meropenem (Table 1).

In addition, the results of failed filter mating experiments suggested that pKP8022_KPC and pKP7709_KPC might be unconjugated plasmids. However, for further evidence of the role of the *bla*_KPC-189_-carrying plasmid in mediating drug resistance, the results of the plasmid transformation experiments illustrated that the MIC of the CZA was 8 mg/L in *bla*_KPC-189_-carrying *E. coli* DH5a_pKP8022_KPC (32-fold increase relative to *E. coli* DH5a, 16-fold increase relative to *E. coli* DH5a_pKP7709_KPC) and the CFDC was 1 mg/L in *E. coli* DH5a_pKP8022_KPC (8-fold increase relative to *E. coli* DH5a, 4-fold increase relative to *E. coli* DH5a_pKP7709_KPC) (Table 1). The results showed that compared with the *bla*_KPC-2_-carrying plasmid pKP7709_KPC, the *bla*_KPC-189_-carrying plasmid pKP8022_KPC mediated resistance to CZA while recovering susceptibility to carbapenem antibiotics.

### Comparison of the ligand binding structures of KPC-2 and KPC-189

**Fig 3:**
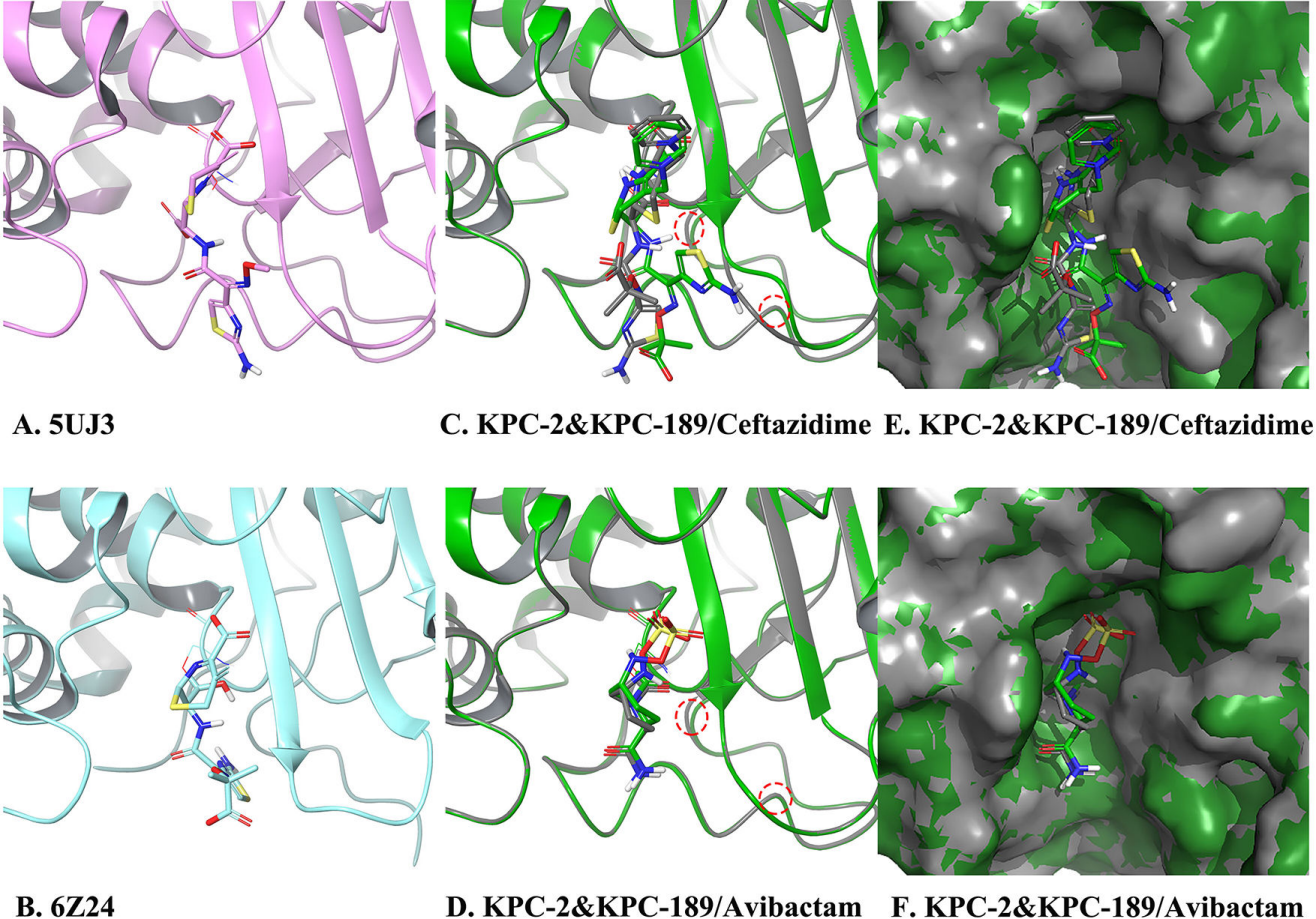
Structural comparison of KPC-2 and KPC-189 complexed with ceftazidime and avibactam. (A–D) Binding mode comparison of KPC-2 and KPC189. (E–F) Receptor surface comparison of KPC-2 and KPC-189. Cartoon representations, ligand carbons, and surfaces are colored according to receptors. Those of 5UJ3 (cefotaxime-bound KPC-2) are colored in pink, those of 6Z24 (ceftazidime-bound KPC-2 E166Q) are colored in cyan, those of ligand-docked KPC-2 are colored in gray, and those of ligand-docked KPC-189 are colored in green. The A172T and D179Y mutations are shown as red dashed circles.

To compare the ligand binding abilities of KPC-2 and KPC-189, ligand-bound models were generated through covalent docking with *ΔG* calculations. All of the scoring results are listed in Table 3. As is well known, *ΔG* represents how tightly ligands bind to KPCs, and the covalent docking affinity (cdock) represents how strongly a forward covalent reaction occurs. The cdock affinities of both ceftazidime and avibactam decreased in KPC-189 compared with KPC-2, and the cdock affinity of ceftazidime decreased more. Thus, compared with that of KPC-2, the ability of KPC-189 to hydrolyze ceftazidime was weaker than its ability to react with avibactam, which indicates that KPC-189 becomes more resistant to ceftazidime–avibactam than KPC-2, as shown by the drug susceptibility tests. Ceftazidime and avibactam both bind to Ser70, Ser130, Asn132, and Thr237 of KPC-2 and KPC-189, according to Fig. 3, while Trp105, Thr235, and Arg220 are also important for ligand binding through numerous hydrogen bonds, salt bridges, and π stacking. All of the bonds help the ligand berth in a precise pose prone to reaction and have been demonstrated multiple times in the PDB database.

**TABLE 3:** Ligand binding ability of KPC-2 and KPC-189

Lactamases	Ligands	*ΔG*_top_pose_ (kcal/mol)	cdock affinity of the top pose
KPC-2	Ceftazidime	−54.441	−6.184
	Avibactam	−31.985	−5.545
KPC-189	Ceftazidime	−53.321	−4.686
	Avibactam	−37.313	−4.586

As shown in Fig. 3, the positions of the isothiazole and pyridine rings profoundly affect ceftazidime binding to KPC mutants, whereas KPC-189 has a deeper reaction center on Ser70 and a shallower binding pocket, especially the lip around the Ω-loop, than KPC-2. As shown in Fig. 3, the D179Y mutation of KPC-189 pushes the Ω-loop up to narrow the pocket lip, while its A172T mutation pushes the β7-β8 loop up more. Thus, the jacked Thr237 attracted the amide group of ceftazidime to the right side, the isothiazole ring was subsequently removed, and the β-lactam ring was removed from the reaction center. It weakens ceftazidime binding to KPC-189 and obstructs its covalent reaction. For a similar reason, the urea bond in the ring of the avibactam was pushed away from Ser70 slightly considering its smaller volume, although its sulfonyl imine group was twisted near Ser70 to squeeze the oxyanion hole and strain the hydrogen bond network in the reaction center, especially near Ser130. This treatment led to a similar decrease in cdock scores but a greater *ΔG* in KPC-189 than in KPC-2, resulting in poorer inhibition efficiency in KPC-189. In general, both ceftazidime and avibactam decrease the binding ability of these strains to KPC-189 compared to that of KPC-2, because the A172T and D179Y mutations, especially A172T, cause shallow changes in the pocket lip and the jack-up β7-β8 loop. Due to its larger volume, the ability of KPC-189 to degrade ceftazidime was greater than its ability to inhibit avibactam. Consequently, KPC-189 was more resistant to the ceftazidime–avibactam combination than KPC-2.

### Characterization of the *bla*_KPC-189_-carrying plasmid

**Fig 4:**
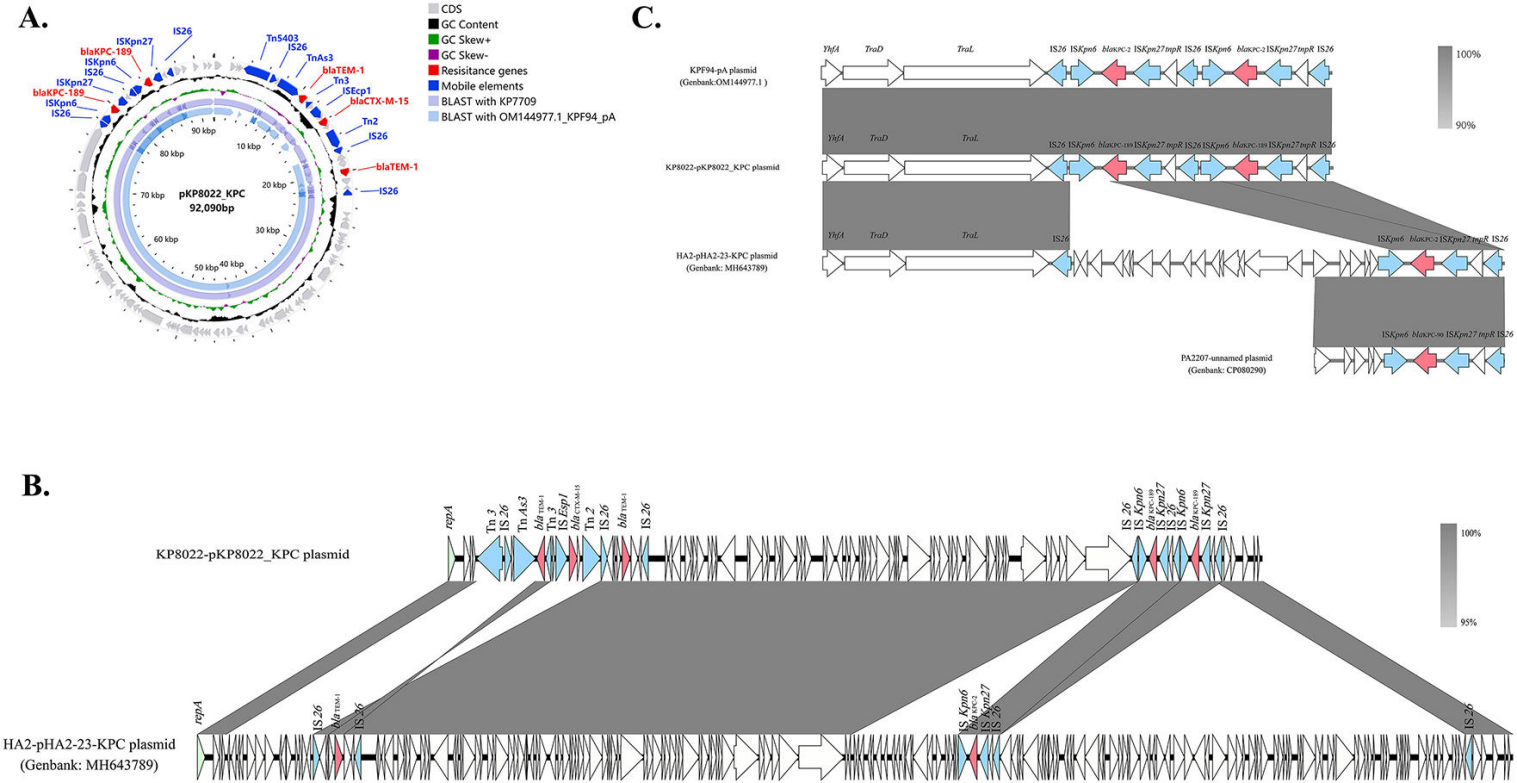
(A) Comparative analysis between the pKP8022_KPC plasmid and other similar plasmids. pKP8022_KPC was used as the reference plasmid for comparison with the KPF94_pA plasmid and the whole genome of KP7709.** (B)** Schematic illustration comparing the structural features of the plasmid pKP8022_KPC with those of the plasmid pHA2-23-KPC (MH643789). (**C)** Genetic context of *bla*_KPC_ on pKP8022_KPC and related plasmid sequences. Gray shading and squares indicate homologies between the corresponding genetic loci on each plasmid. Arrows indicate open reading frames, with arrowheads indicating the direction of transcription: red, antibiotic resistance-encoding genes; blue, transposon- and integron-associated genes; green, replication-associated genes; other genes are shown by white arrows.

Whole-genome sequence analysis revealed that *bla*_KPC-189_ was carried by the IncFII-type plasmid pKP8022_KPC, which is 92,096 bp in size and contains a variety of antibiotic resistance genes (ARGs), including *bla*_TEM-1_ (two copies), *bla*_CTX-M-15_, *rmtB1*, and *bla*_KPC-189_ (two copies) (Fig. 4A). Moreover, analysis of the whole-genome data of strain KP7709 revealed that pKP8022_KPC is highly identical to the IncFII-type plasmid pKP7709_KPC carried by KP7709 (Fig. 4A). BLAST analysis revealed that the plasmid pKP8022_KPC had high coverage with the plasmid pHA2-23-KPC in the NCBI database, which carries *bla*_KPC-2_ isolated from clinical *K. pneumoniae* (Fig. 4B). Sequence analysis revealed that the major difference between the two plasmids was that plasmid pKP8022_KPC lacked a segment of 54,354 bp in size and was surrounded by two IS*26* sequences in the same direction (Fig. 4B).

In addition, the double-copy *bla*_KPC-189_ was carried by the tandem core structure enveloped by IS*26* (Fig. 4C). Sequence comparison revealed that the core structure of IS*Kpn6-bla*_KPC_-IS*Kpn27-tnpR*-IS*26* was identified in the genetic surroundings of both the previously isolated *bla*_KPC-2_ gene and *bla*_KPC-90_ gene (Fig. 4C). We identified an IncFII-type plasmid harboring two copies of *bla*_KPC-2_ in the NCBI database, which was found to show complete identity with the surrounding genetic environment of *bla*_KPC-189_ (10,197 bp) (Fig. 4C). Both plasmids contained two duplicates of the IS*Kpn6-bla*_KPC-_IS*Kpn27-tnpR*-IS*26* core structure and were interspersed with the three-copy IS*26* in the structure surrounding the 10,179 bp region. This tandem core structure apparently evolves *in vivo* during infection, although not by self-transferring, and multiple IS*Kpn27-bla*_KPC_-IS*Kpn6* copy numbers could mediate transferable CZA resistance upon mobilization. So, we speculated that cointegrate formation is mediated by the replication mechanism of IS*26* during the development of strains. The IncFII-type plasmid carried two copies of *bla*_KPC-2_ that could be mutated to the new KPC variant *bla*_KPC-189_ under CZA pressure while mediating CZA resistance. Therefore, the evolutionary trajectory of KP8022 in the development of CZA resistance may include the following: (i) the core structure of IS*Kpn27-bla*_KPC_-IS*Kpn6* carrying single-copy *bla*_KPC-2_; (ii) the self-replication of the *bla*_KPC-2_-carrying core structure mediated by IS*26* to create a tandem core structure with double-copy *bla*_KPC-2_; and (iii) the *bla*_KPC-2_ mutation to *bla*_KPC-189_ mediates CZA resistance in the presence of the CZA environment (Fig. S1).

### Effects of *bla*_KPC_ genes on host fitness

**Fig 5:**
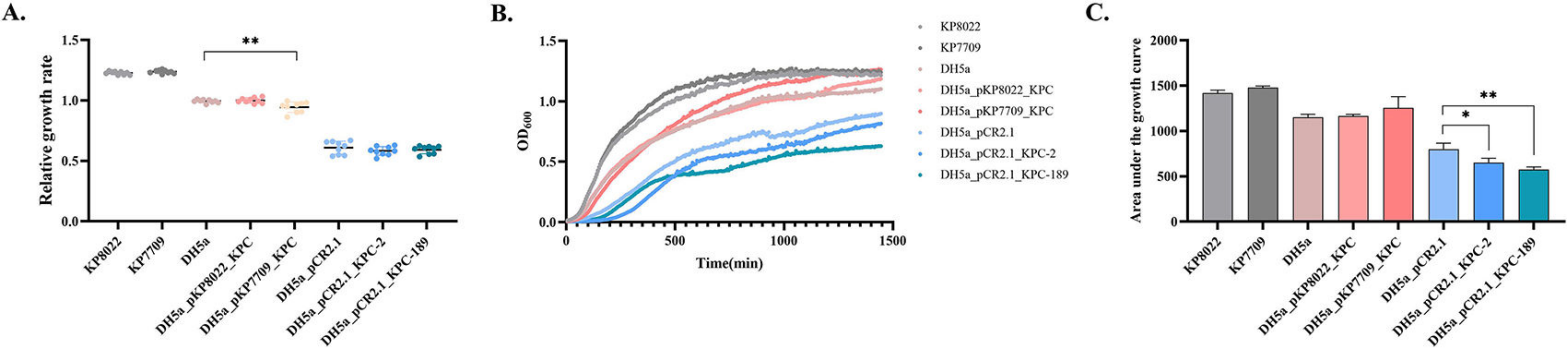
Growth conditions of the constructed strains. The relative growth rate (**A**), growth curve (**B**), and area under the growth curve (**C**) of clinical isolates, transconjugants, and recombinant strains carrying *bla*_KPC_. Representative results of three independent experiments are shown, and error bars represent the standard deviations. *, *P* < 0.05. (unpaired *t* tests).

To investigate the effect of *bla*_KPC_ variants on growth, we determined growth rates without antibiotics using three bacterial growth parameters to reflect fitness [the growth curve, the area under the growth curve (AUC), and the relative growth rate] (Fig. 5A through C). The growth rates of the two clinical isolates KP7709 and KP8022 did not significantly differ (Fig. 5A). The growth rate of *E. coli* DH5a_pKP7709_KPC was lower than that of *E. coli* DH5a by approximately 5%. However, no significant difference between the AUCs was observed, which indicated that compared with the *bla*_KPC-189_-carrying plasmid, the *bla*_KPC-2_-carrying plasmid mainly decelerated the bacterial growth rate (Fig. 5A and C). In addition, we compared the growth rates of cloned strains with different *bla*_KPC_ variants and demonstrated that neither the *E. coli* DH5a_pCR2.1_KPC-2 nor the *E. coli* DH5a_pCR2.1_KPC-189 clones showed significant differences in growth rate relative to the *E. coli* DH5a_pCR2.1 clone; only the AUC significantly decreased (Fig. 5A and C).

## DISCUSSION

CZA is considered an antibiotic with activity against KPC-producing gram-negative bacteria (6). Due to its long-lasting enzyme inhibition effect and low side effects, the widespread use of CZA has greatly facilitated the treatment of KPC-producing CRE infections (17). However, studies from our group have shown that acquired resistance is more likely to develop in patients with a history of CZA treatment (12, 18, 19). In this study, we clarified the evolutionary trajectory of CZA resistance driven by mutations in double-copy *bla*_KPC-2_ to *bla*_KPC-189_ carried by the tandem core structure (IS*Kpn6-bla*_KPC_-IS*Kpn27-tnpR*-IS*26*) during treatment of ST11 CRKP. We hypothesized that the evolutionary trajectory of bacterial populations progressed from *bla*_KPC-2_ single-copy populations to *bla*_KPC-2_ multicopy populations and then *bla*_KPC-189_ multicopy mutant populations, and that IS*26* plays a major role in the formation of tandem multicopy.

One hundred ninety-seven KPC variants were identified in the National Center for Biotechnology Information (NCBI) database through February 2024, and the number of KPC variants increased dramatically in the last 5 years. The result of a core genome phylogenetic tree showed that *bla*_KPC-189_ and *bla*_KPC-95_ were clustered in one clade, and the two variants were found to contain the same mutation at amino acid positions 172 and 179, with the only distinction being that the two variants were mutated from wild-type *bla*_KPC-2_ and *bla*_KPC-3_, respectively (10). At present, NG-test CARBA5 is a routine detection for carbapenemase, which is able to detect and identify the simultaneous production of several of the five main carbapenemases (20). And the NG-test Carba 5 (NG Biotech, Guipry, France) was applied for detecting *bla*_KPC_-carrying bacterial strains in this study. As shown in Fig. S2, our results show positive results for *bla*_KPC-2_ carrying isolates, including the transformant (*E. coli* DH5a_pCR2.1_KPC-2); but negative for transformants carrying *bla*_KPC-189_ (*E. coli* DH5a_pCR2.1_KPC-189). Previous studies showed that D179Y mutation and other mutations in omega loop are associated with negativity of carbapenemase detection methods (21).

In addition, CZA resistance is commonly associated with point mutations in *bla*_KPC_, such as D179Y, V240G, and T234M in CRKP strains (9, 12). The *bla*_KPC-189_ gene contained the combination of the D179Y and A172T mutations within the background of *bla*_KPC-2_, which both occurred in the Ω-loop. The Ω-loop is important because it contains two amino acids (Glu166 and Asn170) involved in the acylation and deacylation of substrates by β-lactamases (13, 14, 22). Previous studies have demonstrated significant threats to broad-spectrum cephalosporins with single amino acid substitutions in the Ω-loop (13, 14, 22). These enzymes are thought to hydrolyze ceftazidime at a greater rate than their parent enzyme, such as the single amino acid substitutions at positions 164, 169, 172, and 179 of SHV-1 and KPC-2, which increased the MIC of ceftazidime in *E. coli* producing these variant enzymes (13, 14, 22). The resistance of these variant enzymes to CZA appears to be caused by increased ceftazidime kinetics and diminished avibactam inhibition (23). However, in this study, the protein structure indicated that the A172T and D179Y mutations in *bla*_KPC-189_ can have a direct effect on the binding affinity of CAZ and AVI for KPC. In general, both ceftazidime and avibactam decrease the binding ability of these strains to KPC-189 compared to that of KPC-2 because the A172T and D179Y mutations, especially A172T, cause shallow changes in the pocket lip and the jack-up β7-β8 loop. Consequently, KPC-189 was more resistant to the ceftazidime–avibactam combination than KPC-2. In addition, this study found that strains harboring KPC-189 decreased CZA and CFDC susceptibility, indicating that these strains developed cross-resistance to CZA and CFDC. Research showed that CFDC mainly synergized with CZA *in vitro* and was effective against both CZA-susceptible and drug-resistant isolates, with a synergy rate of 66.7% (24). The A172T and D179Y substitutions also simultaneously restored the susceptibility of *K. pneumoniae* to carbapenem antibiotics. Previous studies have shown that the D179Y substitution restores the susceptibility of *Enterobacteriaceae* to carbapenems, which is known as the “see-saw” effect (25).

Transposon elements mediating KPC transfer play an important role in KPC genetic variation and evolution (26). A transposable element, Tn*4401*, belonging to the Tn*3* family, was identified as the origin of *bla*_KPC_-like gene acquisition and dissemination, which had the gene order Tn*4401-tnpR*, Tn*4401-tnpA*, IS*Kpn7*, *bla*_KPC_, and IS*Kpn6* (27). Importantly, Tn*4401* has evolved numerous isoforms during subsequent transmission (28). However, KPC variants have various core structures during strain evolution, including IS*Kpn6-bla*_KPC_-IS*Kpn28*, *tnpR-tnpA-*IS*Kpn7-bla*_KPC_-IS*Kpn6*, and IS*Kpn27-bla*_KPC_-IS*Kpn6* (28). Among them, the core structure of *bla*_KPC-189_ is IS*Kpn27-bla*_KPC_-IS*Kpn6*, which is consistent with the surrounding regions of *bla*_KPC-2_ and *bla*_KPC-90_ identified in the NCBI database (GenBank accession numbers MH643789 and CP080290). In addition, IS*26* could mediate cointegrate formation between its host and the target molecule through a replication mechanism (29), which ultimately leads to the formation of a tandem core structure enveloped by IS*26* that carries double copies of *bla*_KPC-189_ (IS*26-*IS*Kpn6-bla*_KPC-189_-IS*Kpn27-tnpR*-IS*26*-IS*Kpn6-bla*_KPC-189_-IS*Kpn27-tnpR*-IS*26*). In addition, double-copy KPC has been shown to be associated with CZA resistance, and the susceptibility to ceftazidime–avibactam was modified due to increased copy number of *bla*_KPC-3_ as a consequence of double-carrying the Tn*4401* transposon (30). However, in isolates with mutated *bla*_KPC_, where mutant *bla*_KPC_ may play a major role in CZA resistance, increased expression and copy number of the mutant *bla*_KPC_ gene may result in high levels of resistance to CZA (31). Therefore, the coexistence of double-copy *bla*_KPC-189_ may play an important role in high-level CZA resistance. In addition, the widespread use of CZA might induce the production of more KPC variants, leading to the emergence of more highly resistant clinical strains. These results indicated that these factors would undoubtedly lead to the further spread of CZA resistance in *K. pneumoniae*, posing a challenge to clinical anti-infective therapy.

The *bla*_KPC_ genes are normally transported by plasmids of different Inc-types, including IncFII, Inc2, and IncN (4, 32, 33). Among these plasmids, IncFII-type plasmids, which generally possess low copy numbers and are distributed across different species of *Enterobacteriaceae,* are more predominant in the group harboring Tn*4401* than in the other incompatibility groups (34). In this study, pKP8022_KPC carrying *bla*_KPC-189_ was identified as an IncFII-type plasmid that carries multiple resistance genes and has no fitness cost to the host. In particular, compared with the wild-type *bla*_KPC-2_ gene, the *bla*_KPC-189_ gene had no negative effect on fitness. The absence of fitness costs might provide greater survival advantages for such strains in the environment as well as a greater ability to spread resistance genes. Therefore, the cost-free effect of *bla*_KPC-189_ on host bacteria contributes to the establishment of CZA resistance mechanisms in CRKP bacterial populations. The susceptibility of KPC-2 to mutation and the emergence of multiple copies will undoubtedly further promote CZA resistance, which poses a considerable challenge for clinical treatment. Thus, CZA drug monitoring and monitoring of changes in clinical sensitivity to antimicrobials of patients is required to optimize efficacy of antibiotics, reduce selection of drug-resistant mutants, and reduce concentration-related adverse effects. In addition, new β-lactam-β-lactamase inhibitor combinations may be used for treating infections caused by KPC variant-producing *Enterobacterales* strains, including meropenem-vaborbactam, imipenem-relebactam, and aztreonam-avibactam; or other antimicrobial agents such as the combination of tigecycline and polymyxin, as well as periodical active surveillance of CZA-R strains with intestinal colonisation (11). In summary, measures must be adopted to prevent the widespread spread of CZA-resistant ST11-type *K. pneumoniae*.

### Conclusion

In this study, we clarified the evolution of resistance driven by mutations in the double-copy gene *bla*_KPC-2_ to *bla*_KPC-189_ during CZA exposure in ST11 CRKP strains. The A172T and D179Y substitutions in KPC-189 can have a direct effect on the binding affinity of CAZ and AVI for KPC. The tandem core structure IS*Kpn27-bla*_KPC_-IS*Kpn6*, which carries a double copy of *bla*_KPC-189_, is surrounded by IS*26*. The IS*26* insertion sequence plays an important role in the formation of the tandem core structure and may lead to further dissemination of CZA resistance. In addition, the *bla*_KPC-189_ gene has no fitness cost, and the possibility that strains are susceptible to CZA-resistant mutations during CZA treatment of *K. pneumoniae* infections should be noted. Therefore, aggressive detection is needed to prevent further transmission of CZA-resistant *K. pneumoniae*.

## MATERIALS AND METHODS

### Patient and isolated data

The patient is an 82-year-old female who was hospitalized in May 2023 for unconsciousness. *K. pneumoniae* KP0047 and KP7709 isolates were isolated from sputum and blood specimens of patients on 20 May and 17 June 2023, respectively. *K. pneumoniae* KP8022 was isolated from urine specimens during CZA treatment (1.25 g iv q8h) on 18 June 2023. The isolates were identified by matrix-assisted laser desorption/ionization time-of-flight mass spectrometry (MALDI-TOF/MS) (bioMérieux, Marcy l’Etoile, France) and further confirmed by whole-genome sequence.

### Antimicrobial susceptibility testing

Antimicrobial susceptibility testing was performed through the broth microdilution method recommended by the Clinical and Laboratory Standards Institute (CLSI) guidelines (35). The antibiotics included cefepime (FEP), ceftazidime (CAZ), ertapenem (ETP), imipenem (IPM), meropenem (MEM), ciprofloxacin (CIP), amikacin (AMK), colistin (CST), tigecycline (TGC), and CZA. MICs of colistin and tigecycline were interpreted in accordance with the European Committee on Antimicrobial Susceptibility Testing (EUCAST) guidelines (36), whereas MICs of the other antibiotics were interpreted according to CLSI M100, 34nd Edition (35). *E. coli* ATCC 25922 was used as a quality control strain.

### Whole-genome sequencing and bioinformatics analysis

The genomic DNA of isolates was extracted using the QIAamp DNA Minikit (Qiagen, Valencia, CA, USA) for whole-genome sequencing using Illumina HiSeq and Nanopore MinION platforms at Zhejiang Tianke (Hangzhou, China). The complete genome sequences were hybridized with the hybridization assembly tool Unicycler 0.4.8 (37) and RAST (http://rast.nmpdr.org) for assembly and gene annotation, respectively. Identification of ARGs was performed using the ResFinder database (38) and Abricate 0.8 (https://github.com/tseemann/abricate). Related sequence comparisons were performed using BLASTn v2.4.0 (39) and visualized by Easyfig v2.2.3 (40). To explore the mechanism of CZA resistance, whole-genome sequences were compared between CZA-sensitive and CZA-resistant isolates using Snippy V4.4.5 (https://github.com/tseemann/snippy) and breseq v0.33.0 (41).

The phylogenetic tree of KPC variants based on single nucleotide polymorphisms (SNPs) was constructed with Snippy-multi (https://github.com/tseemann/snippy) and Fasttree. The tree was further visualized by iTOL (https://itol.embl.de/). And, a core SNPs alignment was generated by snippy-multi using KP8022 as the reference.

### Sequencing depth measurement and real-time quantitative PCR

The number of *bla*_KPC_ copies per cell was assessed using sequencing depth measurements method. Illumina short reads were realigned to map the genome using bwa-mem v.0.7.17 (42). To assess chromosome sequencing depth, single-copy gene regions were selected and their average depth was calculated using sam-Tools v.1.11 (43) with the option “depth-aa.” The *bla*_KPC_ gene copy numbers were expressed as the ratio of gene sequencing depth to chromosome sequencing depth.

To further confirm the copy numbers of the *bla*_KPC_ in two clinical isolates the real-time quantitative PCR (RT-qPCR) was performed, which was calculated as described previously (44). Genomic DNA was extracted using the QIAamp DNA minikit (Qiagen, USA), and the quality and quantity of genomic DNA were determined using a NanoDrop spectrophotometer. RT-qPCR experiment was performed in a LightCycler 480 system (Roche, Switzerland) using TB Green Premix Ex Taq II (TaKaRa Bio). The experiment was completed with a biological triple and a technical triple. Primers are listed in Table S1.

### Cloning experiments

Cloning experiments were performed according to the previous study (45). Briefly, the regions of the *K. pneumoniae* KP7709 and KP8022 genomic sequences containing the open reading frames of *bla*_KPC-2_ and *bla*_KPC-189_, respectively, were cloned into pCR2.1 vectors (Invitrogen, Shanghai, China). The recombinant plasmids pCR2.1_KPC-2 and pCR2.1_KPC-189 were chemically transformed into *E. coli* DH5α strains. The transformants were screened by screening on MH agar plates containing 50 mg/L kanamycin and further verified by PCR and sequencing. Primers used are listed in Table S1.

### Plasmid transformation and filter mating experiments

As previously described (46), *E. coli* J53 was selected as the recipient strain for the filter mating experiments, and the transconjugants were screened on the corresponding Mueller-Hinton (MH) agar plates (for *bla*_KPC-2_-harboring plasmid: 1 mg/L MEM; for *bla*_KPC-189_-harboring plasmid: 1 mg/L CZA. In plasmid transformation experiment, plasmid DNAs of two clinical isolates were obtained by a Qiagen plasmid midi kit (Qiagen, Germany) and chemically transformed into *E. coli* DH5α, respectively (43). MH agar plates containing the corresponding antibiotics were used to select the transformants as well. Finally, the presence of the sequences *bla*_KPC_ and IncFII_*repA* in the transconjugants and transformants was determined by PCR, which was further confirmed by antimicrobial susceptibility testing. Primers used are listed in Table S1.

### Structure modeling and ligand docking

After multiple alignments, the crystal structure of hydrolyzed cefotaxime-bound KPC-2 in *K. pneumoniae* (Protein Data Bank entry: 5UJ3) was employed as the template and mutated into KPC-189 through A172T and D179Y. The residue number and ligands of models were reserved. Then the structures of KPCs were preprocessed and refined using the OPLS3e force field (47) pending ligand preparation for diverse ionization states and isomers. The protein systems were checked afterward to confirm no steric clashes or other abnormalities.

Defining Ser70 as the reactive residue and the ligands in the models as the grid box center, covalent docking (48) was applied to predict the possible binding poses of KPC-2 and KPC-189 in this study using the β-lactam and self-defined diazabicyclooctane reaction types. After docking and refining atoms within 3.0 Å away from the reaction center, the binding free energy using (*ΔG*) was calculated through Prime MM-GBSA (49) for the top output poses of each ligand to compare their binding affinities.

### Growth rate determination

As previously described (50) in order to investigate the growth of the strains in the absence of antibiotics, we performed growth rate measurements of strains including KP8022, KP7709, DH5a_pKP7709_KPC, DH5a_pKP8022_KPC, DH5a_pCR2.1_KPC-2, and DH5a_pCR2.1_KPC-189. The growth rate was calculated using the R script to estimate the growth rate based on the OD_600_ curves as previously described (50), and unpaired *t* tests that returned a *P* value of 0.05 were considered significant.

## Data Availability

The genome sequence of K. pnenmoniae KP0047, KP7709, and KP8022 reported in this study has been deposited in the GenBank nucleotide database under accession nos. JBCGZU000000000, JBAGBV000000000, CP144138, CP144139, CP144140 and CP144141. In addition, the blaKPC-189 sequence was deposited in the NCBI database under accession no. OR501577.

## SUPPLEMENTAL MATERIAL

Fig. S1 msystems.00722-24-s0001.docx Evolutionary roadmap of IncFII plasmids carrying KPC-related core structures in *K. pneumoniae*.

Fig. S2 msystems.00722-24-s0002.docx Fig S2. Characterization of detecting blaKPC.

Table S1 msystems.00722-24-s0003.docx Description of primers used in this study.

ASM does not own the copyrights to Supplemental Material that may be linked to, or accessed through, an article. The authors have granted ASM a non-exclusive, world-wide license to publish the Supplemental Material files. Please contact the corresponding author directly for reuse.
