# Supplementary material for: Evolution of ceftazidime–avibactam resistance driven by mutations in double-copy blaKPC-2 to blaKPC-189 during treatment of ST11 carbapenem-resistant Klebsiella pneumoniae
Source: mSystems. 2024 Sep 17;9(10):e00722-24. doi: 10.1128/msystems.00722-24 (PMC11495026; doi:10.1128/msystems.00722-24)
Supplement: Fig. S1 — Evolutionary roadmap of IncFII plasmids carrying KPC-related core structures in K. pneumoniae. [file msystems.00722-24-s0001.docx]

**
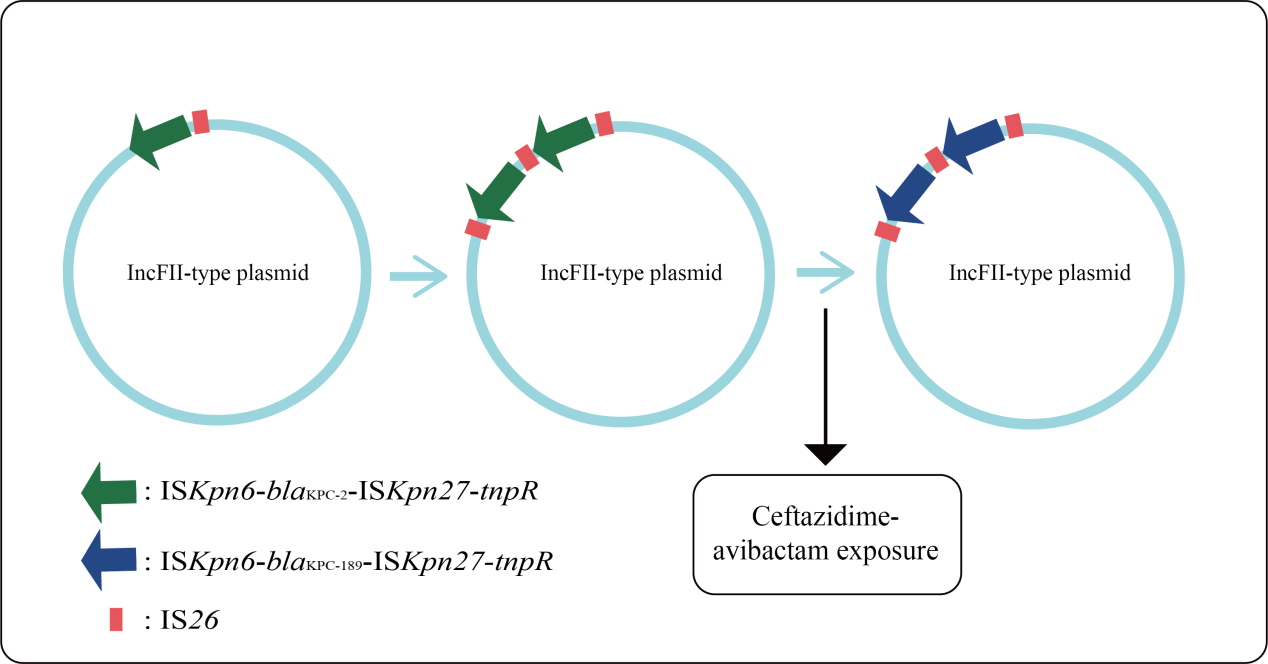
**

**Supplementary figure 1:** Evolutionary roadmap of IncFII plasmids carrying KPC-related core structures in *K. pneumoniae.*
