# Supplementary material for: Evolution of ceftazidime–avibactam resistance driven by mutations in double-copy blaKPC-2 to blaKPC-189 during treatment of ST11 carbapenem-resistant Klebsiella pneumoniae
Source: mSystems. 2024 Sep 17;9(10):e00722-24. doi: 10.1128/msystems.00722-24 (PMC11495026; doi:10.1128/msystems.00722-24)
Supplement: Fig. S2 — Fig S2. Characterization of detecting blaKPC. [file msystems.00722-24-s0002.docx]

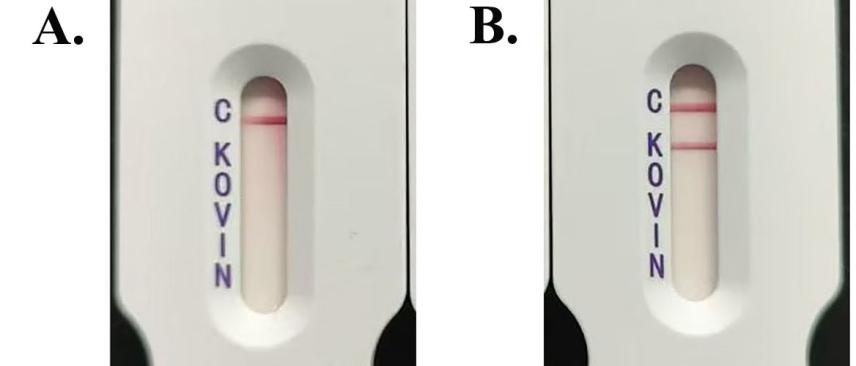


**Supplementary figure 2:** Characterization of detecting *bla*_KPC_. **A.** KPC negative for DH5a_pCR2.1_KPC-189. **B.** KPC positive for DH5a_pCR2.1_KPC-2.
