# Supplementary material for: Evolution of ceftazidime–avibactam resistance driven by mutations in double-copy blaKPC-2 to blaKPC-189 during treatment of ST11 carbapenem-resistant Klebsiella pneumoniae
Source: mSystems. 2024 Sep 17;9(10):e00722-24. doi: 10.1128/msystems.00722-24 (PMC11495026; doi:10.1128/msystems.00722-24)
Supplement: Table S1 — Description of primers used in this study. [file msystems.00722-24-s0003.docx]

**Supplementary table 1. Description of primers used in this study.**

| **Target** | **Primer name** | **Sequence （5'-3')** |
| --- | --- | --- |
| *bla*_KPC_ | KPC_N- F | ATGTCACTGTATCGCCGTCTAGT |
|  | KPC_N- R | TTACTGCCCGTTGACGCCCAA |
| IncFII_*repA* | KP_IncFII_*repA*- F | TTGATTTCGCCATTCATGTG |
|  | KP_IncFII_*repA*- R | GTTACGTGACAGAATCATGC |
| *pgi*(for Qpcr) | Qpcr_*pgi* - F | TTCA TCGCTCCGGCTATCAC |
|  | Qpcr_*pgi* - R | CCGGGTCTTTACCCTGATCG |
| *bla*_KPC_(for Qpcr) | Qpcr-KPC-F | CGCCGTCTAGTTCTGCTGTC |
|  | Qpcr-KPC-R | CCGCCAAAGTCCTGTTCGAG |
| Plasmid backbone of PCR2.1 | PCR2.1-F | CCGCTCGAGCATGCATCTAG |
|  | PCR2.1-R | CCGCCAGTGTGATGGATATC |
| *bla*_KPC-2_ of KP7709 | KP7709-KPC-2-F | GATATCCATCACACTGGCGGaacggtcgtatcagcgacat |
|  | KP7709-KPC-2-R | CTAGATGCATGCTCGAGCGGgtggttggtaatccatgccg |
| *bla*_KPC-189_ of KP8022 | KP8022-KPC-189-F | GATATCCATCACACTGGCGGaacggtcgtatcagcgacat |
|  | KP8022-KPC-189-R | CTAGATGCATGCTCGAGCGGgtggttggtaatccatgccg |
